# US/CT fusion imaging and virtual navigation to guide lumbar intradiscal oxygen-ozone therapy: a pilot study

**DOI:** 10.1007/s40477-023-00835-y

**Published:** 2023-12-15

**Authors:** Domenico Albano, Carmelo Messina, Salvatore Gitto, Stefano Fusco, Luca Maria Sconfienza, Alberto Bellelli

**Affiliations:** 1 IRCCS Istituto Ortopedico Galeazzi, Milan, Italy; 2 Dipartimento di Scienze Biomediche, Chirurgiche ed Odontoiatriche, Università Degli Studi di Milano, Milan, Italy; 3 Dipartimento di Scienze Biomediche per la Salute, Università Degli Studi di Milano, Milan, Italy; 4 Unità Operativa Complessa di Radiologia Diagnostica ed Interventistica, Ospedale Fatebenefratelli San Pietro, Rome, Italy

**Keywords:** Fusion, Spine, Lumbar, Ultrasound, CT, Ozone

## Abstract

**Purpose:**

To test the feasibility of US/CT fusion imaging to guide lumbar intradiscal O_2_/O_3_ therapy to treat discogenic degenerative low back pain due to lumbar disc herniation (LDH).

**Methods:**

We retrospectively included consecutive patients affected by low back pain and/or sciatica due to LDH resistant to conservative therapies, who underwent to lumbar intradiscal O_2_/O_3_ injection under CT/US fusion imaging guidance (Fusion Group) and standard CT guidance (Control Group). For each procedure, we collected procedure operative time, room utilization time, number of CT passes, complications, and O_2_/O_3_ intradiscal diffusion adequacy. Technical success was defined as the ability to complete the procedure as initially planned to reach the disc. Technical efficacy was based on O_2_/O_3_ intradiscal diffusion adequacy, as demonstrated by the last CT scan.

**Results:**

Six patients (4 males; mean age: 68 ± 15 years) were included in the Fusion group, six (4 males; mean age: 66 ± 12 years) in Control group. No complications were observed in both groups. In Fusion group we found significantly lower room utilization time (30 ± 6 min vs. 46 ± 10 min, *p* = 0.008), procedure operative time (14 ± 3 min vs. 24 ± 6 min, *p* = 0.008), and number of CT passes (2 [2,2] vs. 3 [3,3], *p* = 0.006) than in Control Group, respectively. Technical success and efficacy were 100% in both Groups.

**Conclusion:**

CT/US fusion imaging seems to be a feasible and safe guidance for intradiscal O_2_/O_3_ injections, allowing decrease of procedure time and number of CT passes.

## Introduction

Low back pain (LBP) is a quite common condition, particularly in elderly, affecting about 80% of people during their lives [1]. Hence, this condition has a non-negligible social and economic relevance. It is generally related to degenerative changes of the lumbar spine, and it has been associated to lumbar disc herniation (LDH) in 9% of patients [2]. As a matter of say, surgery is considered as the last and less desirable option, although it may become necessary if neurological symptoms occur. LBP is generally first approached with conservative treatments such as anti-inflammatory drugs, physical therapy, life modifications, specific exercises, and weight loss. As a second step, minimally invasive therapies can be provided, mostly including injections around the spine. Among them, an increasing interest and use have been observed over the last years regarding percutaneous injection of oxygen-ozone (O_2_/O_3_) mixtures. These injections may have different targets, including the epidural space, foramina, facets, and intervertebral discs.

The impact of ozone in LDH is thought to be related to its anti-oxidant and anti-inflammatory properties, which can contrast the inflammatory response triggered by LDH as an attempt to decrease the size of the hernia itself. Although controversial results have been reported, recent data support the use of O_2_/O_3_injections for treating LDH, with substantial and significant clinical improvement after treatment, as reported by a recent meta-analysis [3]. Injections can be performed under imaging guidance (CT, fluoroscopy or, less frequently, ultrasound), which ensures needle tip positioning exactly at the specific target [4–8]. Alternatively, injections may be performed without imaging guidance in the muscular paravertebral tissues at the level of pathologic disc. Notably, imaging guidance is essential for an accurate periradicular, intraforaminal, or intradiscal injection. In this setting, a recent meta-analysis has shown that image-guided O_2_/O_3_ mixture injection had better performance for LBP when compared to non-image-guided procedures, with better effectiveness and lower age-related variability in outcomes [4]. Ultrasound (US) guidance has been rarely used for O_2_/O_3_ injections in the spine, for procedures involving foraminal [5] and paravertebral muscles [8] injections. No previous studies focused on the application of US to guide O_2_/O_3_ injections in the lumbar discs, due to deep location of the disc that can be hardly recognized on US.

Over the last years, more and more applications have been introduced for fusion imaging to match real-time US with other more panoramic cross-sectional modalities, such as computed tomography (CT) and magnetic resonance imaging (MRI) [9]. This technology has been improved to integrate US with pre-intervention CT or MRI, making “visible” a target that cannot be visualized on US. Further, needle tracking software may enable following the needle path even when the tip is not directly seen [9]. This technology is routinely used for several procedures involving prostate, liver, and brain [10–12], while in the spine its use has not been extensively reported, with limited studies having proven its feasibility and efficacy for facet and sacroiliac joints injections, and bone biopsies [9, 13, 14]. Fusion imaging guidance has the potential of reducing the number of CT scans that should be acquired to track the needle, and in turn radiation exposure and procedure duration, improving safety and effectiveness of the intervention. Therefore, we carried on a pilot study to test the feasibility of US/CT fusion imaging and virtual navigation in the guidance of lumbar intradiscal O_2_/O_3_ therapy for treating LDH.

## Materials and methods

### Study design

This is a single-center retrospective observational study. Institutional Review Board approval was obtained and informed consent was waived. After matching imaging and clinical data, our database was anonymized to remove any connections between data and patients’ identity according to the General Data Protection Regulation.

We retrospectively included consecutive patients admitted to the Radiology Department of IRCCS Ospedale Galeazzi-Sant'Ambrogio to be subjected to lumbar intradiscal O_2_/O_3_therapy in May 2023. Included patients were affected by LBP and/or sciatica due to LDH resistant to conservative therapies (anti-inflammatory medications, physical therapy, and life changes) and were sent by a referral orthopaedist for lumbar injections. Exclusion criteria were: (i) pregnancy; (ii) coagulation impairment; (iii) motor deficits; (iv) spine infections; (v) neoplastic vertebral lesions; and (vi) calcified disc herniations [15]. Our institutional database was searched for standard CT-guided procedures like those subjected to injections under fusion imaging in terms of age, gender, and disc level. Data from these patients were used as controls. Therefore, patients included in this study were divided in those subjected to standard percutaneous CT-guided lumbar intradiscal O_2_/O_3_ therapy (Control Group) and those treated using US/CT fusion imaging and virtual navigation as guidance for injections (Fusion Group).

### *Standard and CT* + *US fusion-guided injection*

**Fig. 1:**
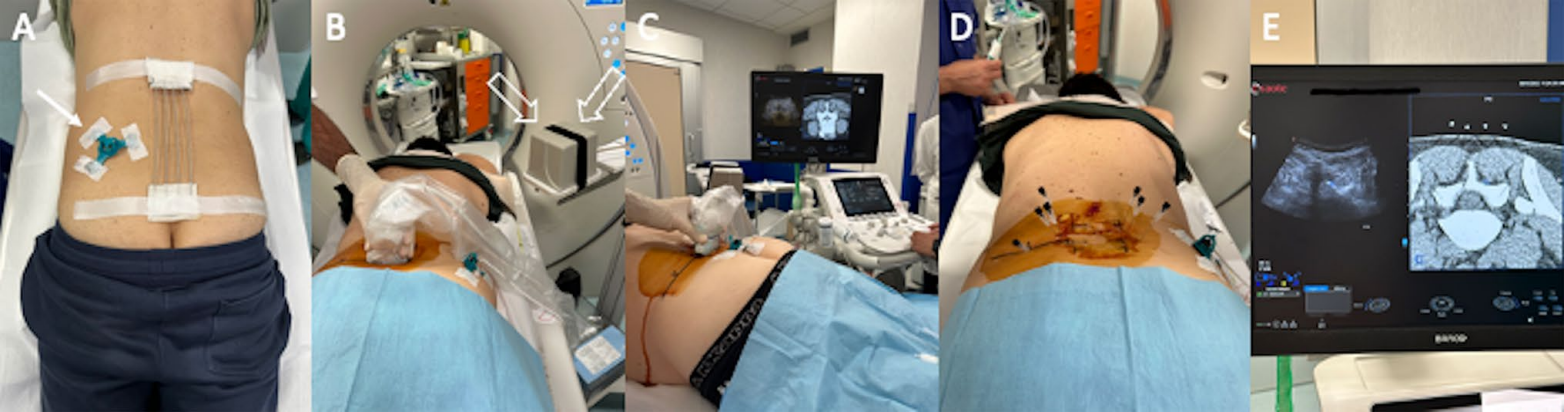
US/CT fusion imaging guided lumbar intradiscal O2-O3 injection. An external fiducial marker was positioned close to the relevant area (arrow, **A**). After disinfection, US probe was placed on the skin to merge images with CT scan (**B**, **C**) that has been already loaded onto the US machine equipped with a GPS-sensor (void arrows, **B**). Six needles were introduced into each facet joint at L3-L4, L4-L5, and L5-S1 levels, and one needle was introduced through a paravertebral oblique postero-lateral in-plane approach to reach the center of the herniated disc (**D**). The navigation system allowed real-time fusion of CT and US image with perfect superimposition of the posterior vertebral arch (**E**)

**Fig. 2:**
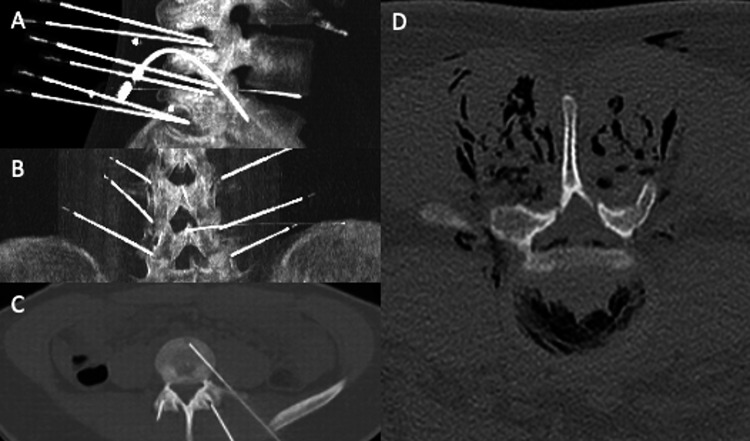
Conventional percutaneous CT-guided injection. The same procedure was done under CT guidance with the needles inserted into the facet joints and into the herniated disc (**A**–**C**). At the end of the procedure, a CT scan was performed to confirm the adequate O_2_/O_3_ intradiscal and paravertebral diffusion (**D**)

Interventions were performed in the CT suite by one musculoskeletal radiologist with 30 years of experience in intradiscal injections and by one musculoskeletal radiologist with 7 years of experience in interventional procedures but without experience in intradiscal injections, although under the supervision of the experienced operator. The coagulation profile was evaluated within 48 h of the procedure and anticoagulants/antiplatelet agents were discontinued, according to the guidelines by the Cardiovascular and Interventional Radiological Society of Europe [16]. Intradiscal O_2_/O_3_ injections were performed with patients under conscious sedation with sterile technique. Patients were placed prone on the CT table (Canon Aquilion, Canon Medical Systems, USA) with a pillow under the belly to increase the lumbosacral angle. An external fiducial marker was positioned in the relevant area and a CT scan of the area of interest was obtained and used as a reference image for planning the needle path and to be matched with US for fusion imaging. DICOM dataset was then loaded onto an US machine equipped with a Global Positioning System (GPS)-based electromagnetic navigation unit, a sensor on the US transducer, and a needle-tracking system (MyLab X90, Esaote, Genova, Italy). The system provides an automatic match of CT images with US transducer and patient positioning with respect to the GPS sensor. Then, skin disinfection and sterile draping were done. Needle tracking was obtained with a single sensor (V-TRAX, Civco Medical Solutions, Coralville, USA), which was directly connected to the needle (15 cm length spinal needle, 22 G). The navigation system enables to monitor real-time the procedure by following a virtual needle that is displayed on either CT and US images. The needle was introduced through a paravertebral oblique postero-lateral in-plane approach to reach the center of the herniated disc (Fig. 1). Then, 20 mL O_2_/O_3_ was injected into the disc. The procedure was completed with periarticular injection of a mixture of local anesthetic (0.5 ml of mepivacaine 1%), steroid (0.3 ml of triamcinolone acetonide), and 10 ml of O_2_/O_3_ into each facet joint at L3-L4, L4-L5, and L5-S1 levels. Last, a CT scan was performed to evaluate the adequate O_2_/O_3_ intradiscal and paravertebral diffusion. All procedures were performed as outpatient, and all patients were advised to return to normal activities after 5 − 7 days, with specific instruction for avoiding bending, and weightlifting for 2 weeks. Pain medications and antibiotic therapy were not prescribed on discharge. Conventional percutaneous CT-guided injections were performed in the same way, but using CT as a guidance only (Fig. 2).

### Data collection, sample size calculation, and statistical analysis

For each procedure, we collected the following data: (i) elapsed time from first CT scan and last injection (procedure operative time); (ii) room utilization time; (iii) number of CT passes; (iv) complications; (v) O_2_/O_3_ intradiscal diffusion adequacy. For what concerns the assessment of intervention duration, we could evaluate the elapsed time from first CT scan to last injection just looking at the time of acquisition of the first and last CT images. Regarding room utilization time, we routinely collect admission and discharge time of our patients from the interventional and CT units in our internal report.

Technical success was defined as the ability to complete the procedure as initially planned [17, 18] to reach the disc. Technical efficacy was based O_2_/O_3_ intradiscal diffusion adequacy, as demonstrated by the last CT scan. Differences in terms of demographics, disc levels, and procedure details were investigated between Fusion Group and Control Group.

Anonymous data were analyzed using MATLAB 2016b (The *MathWorks*, Natick, 2016). Differences among variables were evaluated by Fisher’s exact test for categorical variables, and by non-parametric Mann–Whitney *U*-test for continuous variables. A *p* value < 0.05 was considered to indicate statistical significance. Categorical variables were reported as absolute value and percentage; continuous variables were reported as either mean ± standard deviation or median and interquartile [25th–75th] range.

## Results

Six patients (4 males, 2 females; mean age: 68 ± 15 years) were included in the Fusion Group and six patients (4 males, 2 females; mean age: 66 ± 12 years) were included in the Control Group. LDH involved the L3-L4 level (*n = *1), L4-L5 level (*n = *3), and L5-S1 level (*n = *2) in the Fusion Group, the L3-L4 level (*n = *1), L4-L5 level (*n = *2), and L5-S1 level (*n = *3) in Control Group. No significant differences in age (*p* = 0.749) and gender (*p* > 0.999) were found between the two groups. No complications were observed in both Groups. Room utilization time, procedure operative time, and number of CT passes were 30 ± 6 min, 14 ± 3 min, and 2 [2, 2] in the Fusion group, and 46 ± 10 min, 24 ± 6 min, and 3 [3, 3] in the Control group, respectively. Comparing the two groups, we observed significantly lower room utilization time (*p* = 0.008), procedure operative time (*p* = 0.008), and number of CT passes (*p* = 0.006) in the Fusion group. Patient characteristics and procedure details are resumed in Table 1. Technical success was 100% in both Groups, as the center of the herniated disc was reached with the tip of the needle in all patients. Technical efficacy was 100% in both Groups, as O_2_/O_3_ intradiscal diffusion was adequate in all cases as demonstrated by the last CT scan.

**Table 1:** Patient characteristics and procedure details. Note- bold data are statistically significant

	Fusion (*n = *6)	Controls (*n = *6)	*p*
Age, mean (range)	68 ± 15 years (45–83)	66 ± 12 years (48–81)	0.749
Gender	4 males, 2 females	4 males, 2 females	** > 0.999**
Technical success	100%	100%	
Technical efficacy	100%	100%	
CT passes, median [IQR: 25th–75th]	2 [2–2]	3 [3–3]	**0.006**
Room utilization time, mean (range)	30 ± 6 min (22–39)	46 ± 10 min (33–61)	**0.008**
Procedure operative time, mean (range)	14 ± 3 min (10–18)	24 ± 6 min (16–34)	**0.008**

## Discussion

Our main finding is that CT/US fusion imaging and virtual navigation system seems to be feasible and safe to guide lumbar intradiscal O_2_/O_3_ therapy, with 100% technical success and efficacy. CT/US fusion-imaging guidance ensured a decrease in procedure time and number of CT passes when compared to standard CT-guided interventions.

As abovementioned, the results of recent studies support the use of O_2_/O_3_ therapy for treating LBP and sciatica related to LDH [3]. When intradiscal is injected, imaging guidance must be used to reach the center of the herniated disc and to obtain better clinical improvements [4]. CT and fluoroscopy have been used for guiding this kind of intervention, but a non-negligible amount of ionizing radiations is administered to patients, which is particularly relevant when dealing with young adults. Unfortunately, the target of intradiscal injection cannot be seen directly through US. For this reason, CT/US fusion imaging could be helpful to overcome the limitations of the single imaging modalities used as guidance for interventions. As a matter of fact, CT images must be acquired repeatedly during a CT-guided intervention, with a subsequent increase of radiation exposure [19]. CT/US fusion imaging systems, match preoperative CT images with real-time US images, so that any structure detected on US can be immediately recognized on the corresponding structure on CT image without acquiring additional CT images. Hence, there is no need to stop the intervention to acquire CT images to monitor step-by-step the advancement of the needle that can be followed through fusion imaging guidance, thereby decreasing procedure time and making the intervention well-tolerated by the patient [20]. Last, this virtual navigation system makes spine interventional procedures safe [9]. These important points are all supported by our results. Indeed, we have reported a significant reduction of room utilization time and procedure operative time, that means an improvement of cost-effectiveness of these injections. In addition, the decrease of number of CT passes may help reducing radiation exposure of patients subjected to spine injections. In fact, during procedures guided by CT/US fusion imaging, we needed just pre-operative CT scan and post-operative CT images to confirm the correct placement of the needle, with a total of 2 CT passes for each procedure.

Similar results have been reported by a recent study that compared procedure duration time and clinical outcome of fluoroscopy guided vs CT guided intradiscal O_2_/O_3_ injections. Authors found decreased room utilization time and procedure operative time using fluoroscopy, with similar clinical success at 1-month, highlighting the importance of reducing time consumption [6]. No other studies have investigated the potential application of CT/US fusion imaging for guiding intradiscal injections. Nevertheless, in a similar setting, CT/US fusion has been shown to be helpful in decreasing radiation exposure and making shorter procedure duration time of spine bone biopsies [9]. Notably, during a bone biopsy, the needle must be placed exactly within the target lesion, paying attention particularly when dealing with small lesions. Hence, standalone fusion imaging cannot yet be employed without verifying needle position by CT before biopsy is performed. Conversely, during intradiscal O_2_/O_3_ injection, the operator clearly feels an increase of tissue resistance due to the needle transition when the needle penetrates the disc, with a completely different resistance compared to bon margins of vertebral bodies. Therefore, the correct placement of the needle can be easily perceived by the operator. Of note, in our study, three CT/US fusion imaging guided procedures were performed by a radiologist with 30 years of experience in intradiscal injections and three procedures were done by a radiologist without experience in intradiscal injections, but in all cases 100% technical success and efficacy were reached with two CT passes.

Some limitations of this study must be pointed out. First, the small sample size of our cohort that makes this series just a pilot study to understand the safety and feasibility of CT/US fusion imaging for guiding intradiscal injections. However, statistical significance was obtained for the two most important outcomes. Indeed, we think this is just a starting point for future studies aimed at confirming our results and investigating clinical impact and actual cost-effectiveness of this procedure. In this regard, it should be underlined that recommendations and guidelines have been published about image-guided musculoskeletal interventional procedures performed in the upper [21–23] and lower limb [24–27], but there is still a lack of standardization for what concerns the application of imaging as a guidance in the spine, despite several studies have been published on this topic [28, 29]. Last, we did not directly measure the reduction of radiation dose administered to the patients, but this is witnessed by the reduced number of CT passes that were done to complete fusion imaging guided injections. However, a reduction of the number of CT passes directly implies a reduction of radiation dose usage.

In conclusion, CT/US fusion imaging seems to be a feasible and safe guidance for intradiscal O_2_/O_3_ injections. US fusion with previously acquired CT images ensures real-time needle monitoring and correct needle placement into the herniated disc, allowing decrease of procedure time and number of CT passes needed to perform the injections, regardless operator’s experience. It may improve cost-effectiveness of intradiscal injections, which deserves further investigation by larger prospective studies.

## Data Availability

The datasets used and analyzed during the current study are available from the corresponding author on reasonable request.
